# Cross-Task Differences in Frontocentral Cortical Activations for Dynamic Balance in Neurotypical Adults

**DOI:** 10.3390/s24206645

**Published:** 2024-10-15

**Authors:** Robert D. Magruder, Komal K. Kukkar, Jose L. Contreras-Vidal, Pranav J. Parikh

**Affiliations:** 1 Department of Biomedical Engineering, Carnegie Mellon University, Pittsburgh, PA 15213, USA; 2 Center for Neuromotor and Biomechanics Research, Department of Health and Human Performance, University of Houston, Houston, TX 77204, USA; kkkukkar@cougarnet.uh.edu; 3 Laboratory for Noninvasive Brain-Machine Interface Systems, Department of Electrical and Computer Engineering, University of Houston, Houston, TX 77204, USA; jlcontreras-vidal@uh.edu; 4 IUCRC BRAIN, University of Houston, Houston, TX 77204, USA

**Keywords:** balance, perturbation, sway reference, supplementary motor area, cTBS, EEG

## Abstract

Although significant progress has been made in understanding the cortical correlates underlying balance control, these studies focused on a single task, limiting the ability to generalize the findings. Different balance tasks may elicit cortical activations in the same regions but show different levels of activation because of distinct underlying mechanisms. In this study, twenty young, neurotypical adults were instructed to maintain standing balance while the standing support surface was either translated or rotated. The differences in cortical activations in the frontocentral region between these two widely used tasks were examined using electroencephalography (EEG). Additionally, the study investigated whether transcranial magnetic stimulation could modulate these cortical activations during the platform translation task. Higher delta and lower alpha relative power were found over the frontocentral region during the platform translation task when compared to the platform rotation task, suggesting greater engagement of attentional and sensory integration resources for the former. Continuous theta burst stimulation over the supplementary motor area significantly reduced delta activity in the frontocentral region but did not alter alpha activity during the platform translation task. The results provide a direct comparison of neural activations between two commonly used balance tasks and are expected to lay a strong foundation for designing neurointerventions for balance improvements with effects generalizable across multiple balance scenarios.

## 1. Introduction

The control of balance requires a complex interplay between sensory information arising from visual, vestibular, and somatosensory systems for the generation of motor commands to maintain an upright stance [1]. Balance control predicts the function and engagement of individuals in activities of daily living [2,3,4]. Poor balance control, as it happens due to aging or neurological disorders, is a predictor of falls [5,6,7,8]. For neurotypical adults, standing balance is a seemingly trivial task, controlled primarily by subcortical brain structures and the spinal cord with very limited cortical involvement [9]. Several studies have suggested the involvement of the cortex [10] when the stance is challenged through blindfolding the participant [11], using single support [12,13], standing on an unstable platform [14], or standing on a platform capable of making unexpected translations or rotations [15].

Activations across several fronto-parietal regions [16,17,18,19,20], including anterior cingulate, supplementary motor area (SMA), and posterior parietal cortices [21,22,23,24], are commonly reported, yet causal relationships are not completely understood [25]. Similar tasks often report the same regions of interest but may demonstrate different activation levels. Researchers investigating postural stability usually use a force plate capable of administering a translation of the standing platform (perturbation task) or a force plate that is sway-referenced (sway reference task) in reference to deviations in the subject’s center of pressure [26]. The perturbation task (PT) is associated with characteristic evoked potentials observed in the electroencephalographic (EEG) activity following the perturbation but preceding the initiation of a functional balance task [27]. These perturbation-evoked potentials originate from frontocentral brain regions such as the anterior cingulate or SMA [21,22]. The characteristics of these potentials, such as the amplitude and latency, are known to be affected by the novelty [24], predictability [28,29], attention [30], and speed [31] of the perturbation, observable on a single trial basis [24,32], and they precede postural instability and stepping to prevent imminent falls [23]. For the sway reference task (SRT), on the other hand, these evoked potentials are smaller in magnitude but can be identified during the task [33]. PT and SRT differ in fundamental mechanisms underlying balance control [34]. Platform translation is an imposed mechanical perturbation that can be considered an error signal unrelated to the ongoing stance. However, sway-referencing the platform results in sensory manipulation and alters sensory feedback regarding motor outputs during ongoing control such that their effects are a function of the action of the participant. A smaller evoked response during SRT, when compared with PT, suggests that the cortical response is stronger in the case of PT. None of the studies have performed a comparison of frontocentral cortical activation levels between the PT and SRT tasks in the same group of individuals. Understanding similarities and differences in cortical involvement for these balance tasks will help identify a suitable cortical target for neurorehabilitation strategies for balance improvements. Targeting a common mechanism is likely to have effects that generalize across tasks.

Furthermore, understanding the certainty of the causal influence of cortical activation on the control of balance during challenging conditions is important for rehabilitation researchers. High-frequency repetitive transcranial magnetic stimulation (cTBS) has been used for inducing virtual lesions of brain regions and understanding its effect on balance control [35]. cTBS over SMA in the dominant hemisphere altered EEG band power in lower-frequency bands within brain areas involved in the control of balance during the sway referencing task [18]. However, it is unclear if similar effects will be obtained for another balance task with distinct underlying mechanisms.

This study investigated differences in cortical activation measured using EEG across two commonly used balance tasks, the perturbation task and the sway reference task in neurotypical adults. We expected common EEG sources of brain activation across PT and SRT tasks but differences in their activation levels. Considering the known disruptive effects of cTBS over SMA on rhythmic synchronous cortical oscillations measured non-invasively using EEG during the SRT task, we expected that cTBS over SMA would alter EEG band power within the frontocentral brain regions during PT in neurotypical adults.

## 2. Materials and Methods

Twenty healthy, right-handed young adults (age: 26.0 ± 3.4 years; 8 females; height: 168.0 ± 11.8 cm; weight: 66.7 ± 13.8 kg, mean ± standard deviation (SD)) provided written, informed consent for participation. Two groups (*n* = 10, 4 females) were randomly selected to receive cTBS over SMA (cTBS_SMA_) or a sham control (cTBS_SHAM_). There was no significant difference in age, weight, and height (all *p* values > 0.05) between groups [18]. No participant reported a history of balance, neurological, musculoskeletal, cardiovascular, or vestibular disorders; all participants were screened with the Physical Activity Readiness Questionnaire (PAR-Q) and TMS Adult Safety Screening Questionnaire. The Committee for the Protection of Human Subjects at the University of Houston approved this study.

### 2.1. Instrumentation

#### 2.1.1. Electroencephalography (EEG)

Whole-scalp EEG with 64 active channel electrodes (Brain Products GmbH, Gilching, Germany; 1000 Hz) was recorded at rest and during the balance tasks. An elastic cap constrained the movement of the electrodes during the balance task. A modified international 10-20 system was used, where GND and REF were attached to the earlobes and replaced by T7 and T8 electrodes. However, four electrodes were repurposed for electrooculography (EOG), where the electrodes were placed around the participants’ eyes to remove eye artifacts during EEG preprocessing [18,31].

#### 2.1.2. Computerized Dynamic Posturography (CDP)

A standard, commercial CDP force platform (NeuroCom Balance Manager, Natus Medical Incorporated, Pleasanton, CA, USA) was used for both balance tasks, as conducted previously [18,31]. During all posture tasks, subjects wore a safety harness to prevent falls or injury. The platform is equipped with a dynamic 45.72 cm × 45.72 cm dual-force plate system. The ground reaction forces from under the feet of subjects were collected by four individual force transducers embedded within the force plate. Force platform data were collected at 100 Hz and processed by pre-installed software on a Windows-based desktop connected to the NeuroCom Balance Manager (Research module, NeuroCom software version 8.0, Natus Medical Incorporated, Pleasanton, CA, USA). Our previous work has reported the analysis of the center pressure data during PT [31] and SRT [18]. An analog output signal of 5 V generated by the NeuroCom system was used to synchronize NeuroCom data with the EEG system [31].

### 2.2. Balance Tasks

#### 2.2.1. Sway Reference Task (SRT)

Participants were instructed to perform the sway reference task with varying sensory conditions. The goal of this task was to maintain a quiet, upright stance with eyes closed. They completed nine trials of 20 s duration each, where the platform was referenced to their sway with different gain settings. The orientation of the platform of the balance manager was adjusted to the gravitational vertical by rotating it in the sagittal plane about an axis through the subject’s ankle joint in some proportion (a preselected gain between −2 and +2) to the postural sway of the subject. The purpose of this task was to alter the relationships between postural sway and somatosensory inputs by randomly varying the gain of the support surface. A range of gains (−1.0, −0.4, 0, 0.4, 0.6, 1.0, 2.0) was used to expose subjects to different levels of postural difficulty. Subjects were not informed when the gain changed. These postural conditions allowed us to manipulate the difficulty of the postural control task progressively while concurrently monitoring variations in the EEG responses. Subjects were not allowed to practice at different gains of the sway-referenced support surface. The trials were continuous with a brief (<10 s) delay to save the data and begin the new trial, thus demarcating the start of each trial in the EEG and used in the analysis.

#### 2.2.2. Perturbation Task (PT)

Participants were instructed to maintain an upright stance with eyes closed during destabilizing postural perturbations. The perturbations were in the form of unexpected translations of the force platform upon which the subject stood. Six different perturbation conditions [31] were used, with direction (backward and forward), displacement (3.17 cm and 6.35 cm), speed (7.93 cm/s and 15.88 cm/s), and period (400 ms and 800 ms) varying from condition to condition, randomized in order. Participants completed a total of eighteen discrete trials, three trials per perturbation condition. Each trial lasted for 5000 ms, which included 1000 ms of “pre-perturbation phase,” 400 or 800 ms of “perturbation phase,” and 3600 or 3200 ms of “post-perturbation phase.” In the post-perturbation phase, the force plate remained stationary at the translated position. The onset of perturbation occurred 1000 ms after the trial onset. Participants were not informed of the start of each trial nor warned of perturbation onset. At the end of each trial, the platform translated back to the original position at 1.00 cm/s, which took 3500–6200 ms, and then the next trial was initiated after a brief (<10 s) delay to save the trial data. Due to fewer trials per condition, the analysis grouped all trials to obtain an average frontocentral EEG response to perturbation across conditions and examined whether inhibitory rTMS alters this response. To capture a naïve response, no practice session was provided before the experimental trials. EEG activity was recorded for all the trials.

### 2.3. Transcranial Magnetic Stimulation Procedures

Active motor threshold (aMT) was estimated by placing a figure-of-eight TMS coil (Magstim Super Rapid^2^ stimulator; Magstim, Whitland, UK) tangential to the scalp, oriented at 45° from the midsagittal line, with the handle pointing backward, inducing a current in the posteroanterior direction. The hand motor region of the left primary motor cortex (M1) that is associated with the right first dorsal interosseus (FDI) muscle was located using supra-threshold TMS pulses [36,37]. Electromyographic (EMG) activity in the FDI muscle of the right hand was recorded using a differential surface electrode (Bagnoli EMG system, Delsys, Natick, MA, USA). The largest grip force exerted using the index finger and thumb on an instrumented force-sensing handle over three trials was used as the maximum voluntary force (MVF) [38]. The aMT was determined as the TMS intensity that induced 200 µV peak-to-peak motor evoked potentials (MEPs) in 5 of 10 trials in the FDI muscle during grip force exertion at 20% of MVF [39], using visual feedback. The aMT was estimated to be 52 ± 5% (mean ± SD) of the maximum stimulator output [18].

A 3T Siemens Trio whole-body MR scanner (Erlangen, Germany) produced high-resolution T1-weighted structural images for each subject [18] before the experiment. A three-dimensional (3D) brain was reconstructed from the MRI slices to display the cortical surface (Brainsight software, version 2.1, Rogue Research, Montreal, QC, Canada). The location of the left SMA was demarcated for each subject. Left SMA was selected because of postulated functional asymmetry between motor areas, with the dominant brain hemisphere playing a more important role in the selection of appropriate postural strategies [18]. The location of the left SMA was selected as the most medial part of the superior frontal gyrus, which was anterior and dorsal to the precentral gyrus [40,41,42]. Because SMA is located directly anterior to the leg representation of M1 at the same depth on the interhemispheric surface [43,44], the optimal coil position to evoke MEPs in the right tibialis anterior (TA) muscle was determined first while subjects were instructed to exert ~20% of maximum voluntary contraction of right TA [41,45]. The coil was then moved anteriorly in small increments, and the final SMA location was chosen 1 cm anterior from the point where there were no MEPs in the right TA. If needed, the coil position was slightly adjusted based on the anatomical target. For the virtual lesion, the coil was placed and maintained horizontally with the handle pointing rightward [44]. The Montreal Neurological Institute (MNI) coordinates of the stimulation site for the left SMA were −4.36 ± 2.61, −5.14 ± 1.88, 64.43 ± 4.04 mm (x, y, z, mean ± SD; *n* = 20) [18] and were consistent with those reported in the literature [40,41,46,47,48].

To disrupt the SMA activity, continuous theta burst stimulation (cTBS) was delivered at 80% of aMT of FDI (cTBS_SMA_; *n* = 10). Repetitive cTBS pulses were delivered in the form of bursts of three pulses at 50 Hz at a rate of 5 Hz (a total of 600 pulses) [49]. For the SHAM group (cTBS_SHAM_; *n* = 10), the same stimulation parameters were used, but the coil was placed perpendicularly over the SMA region such that no relevant current flow was induced in the cortical tissue [50,51,52,53]. The reduced corticospinal excitability and altered EEG measures last about 60 min [49] and 30 min [54,55], respectively.

### 2.4. Experimental Procedures

**Figure 1:**
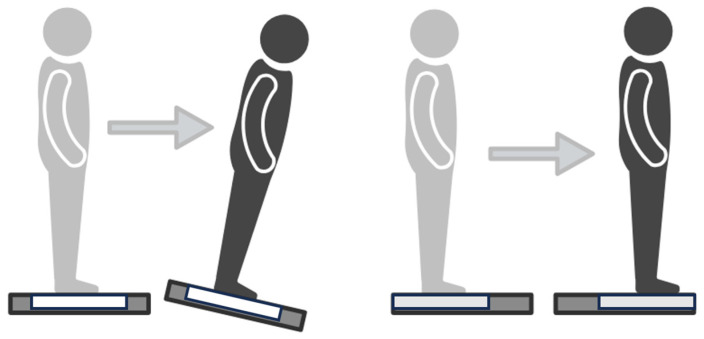
Depiction of each task. (**Left**) Sway reference task, where the platform tilts in reference to the participants’ center of pressure. (**Right**) Perturbation task, where the participant is translated unexpectedly forward. Figure created with BioRender.com (accessed on 15 March 2024).

Each subject participated in two sessions separated by ~14 days. In the first session, participants underwent a structural MRI of the brain, a general assessment of the body, and familiarization trials with the force platform. The second session collected data using EEG, cTBS, and the two balance tasks. Participants were instrumented with EEG, followed by an assessment of the resting state EEG activity. Then, the cTBS procedures, as detailed above, were conducted. After which, the resting state EEG activity was reassessed. All participants performed SRT, followed by PT (Figure 1). Following the two balance tasks, the resting-state EEG was reassessed. Each resting-state collection lasted for 2 min with eyes closed and quiet standing, and the findings have been reported in our earlier work [18]. All procedures (including post tasks resting state EEG) were completed within 27.2 (±1.4) minutes following cTBS.

### 2.5. Data Analysis

#### 2.5.1. EEG Preprocessing

All 64 channels passed through a zero-phase notch filter (60 Hz) to remove line noise and a zero-phase bandpass filter (4th order, 0.1 Hz to 100 Hz, Butterworth). The electrooculography (EOG) channels were used in an H-Infinity filter (q = 1 × 10^−11^, gamma = 1.15) to remove eye artifacts while retaining brain signals [56]. The remaining 60 EEG channels were re-referenced to the common average reference. Artifact subspace reconstruction (ASR) was applied [57] with a cutoff parameter of 30 [58]. ASR utilized a clean EEG signal with a sliding window of 500 ms to denoise the signal. Twenty-second epochs, starting with trial onset, were used for SRT, and 1 s before and 3 s after perturbation onset was used to epoch PT. The potential sources of the cleaned EEG signal were determined through independent components analysis (ICA) with principal component analysis (PCA). DIPFIT in EEGLAB was used to fit dipoles to the provided independent components (ICs). ICA assumes the number of ICs to be equivalent to the number of given channels, but some ICs were removed due to their dipoles being outside of EEGLAB’s boundary element model (BEM) or ICs that were primarily noisy due to channel, muscular, ocular, or bundle artifacts, determined through visual inspection.

#### 2.5.2. EEG Source Localization and EEG Power

A k-means clustering algorithm, with a k value of 5 [18], was applied to the remaining ICs from both SRT and PT tasks within each group based on similarities in calculated features such as dipole 3D location. Only ICs that accounted for at least 85% of variance (less than 15% residual variance) and within 3 standard deviations of the cluster centroid were kept for source localization. The IC that accounted for most of the variance was selected for each participant in each cluster. The Brodmann Areas (BA) for each cluster were determined using the Yale Bioimaging Suite [59] and a deviation of +/−5 mm of the cluster centroids’ Talairach coordinates. Clustering was computed using dipoles for cTBS_SMA_ from the SRT, from the PT, and combined tasks (CT) and repeated for cTBS_SHAM_. All clusters included independent components from at least 60% of subjects suggesting the clusters were representative of the tasks and most of the participants. The region of interest was the frontocentral region, of which both groups had one cluster centroid during CT clustering, and these were used for further analysis. These CT clusters had over 80% of subjects contributing during both PT and SRT.

The power spectrum was calculated for each independent component using the pwelch function in MATLAB (MATLAB 2022a, Mathworks, Natick, MA, USA) with non-overlapping Hamming windows with the default frequency resolution of *π*/256 rad/sample. Relative power for delta (1–4 Hz), theta (4–8 Hz), alpha (8–12 Hz), beta (12–30 Hz), and low gamma (30–50 Hz) frequency bands were calculated by summing the power over each frequency band and normalized with respect to the total power of the PSD (from 1–50 Hz) for each independent component.

#### 2.5.3. cTBS over SMA and the Frontocentral Brain Response during PT

Because the secondary aim was to investigate the effects of cTBS over SMA on the EEG activity in the frontocentral region, an independent component (IC) over the frontocentral region was selected by examining both the two-dimensional topoplots of all the ICs remaining after EEG data cleaning and the corresponding dipole projections in three-dimensions for each subject [31]. A single IC was chosen in the frontocentral brain region for each subject [23]. If the frontocentral response was unclear or distributed between a couple of ICs, of these, the IC that accounted for the largest amount of data variance was selected [23]. The centroid of the 10 equivalent dipoles, 1 per subject, was determined through a single k-means clustering approach, separately for each group. To understand the effects of cTBS on the frontocentral region, the relative power was computed and compared for each subject (individual ICs, as described above) between SMA and sham groups.

### 2.6. Statistical Analyses

Maximum likelihood linear models were used because this approach avoids restrictive assumptions of other approaches, such as repeated measures ANOVA, and accommodates potential missing values. The clustering step identified EEG sources with different locations across groups; thus, separate linear mixed models were built for each cluster within each group (cTBS_SMA_ and cTBS_SHAM_) to investigate differences in EEG source power between SRT and PT. Linear mixed models with within-subject factor Task (SRT, PT), within-subject factor frequency band (delta, theta, alpha, beta, and low gamma), and interaction between task and frequency bands were included in the model.

Next, the effects of cTBS over SMA on EEG source power over the frontocentral region were studied. For EEG frontocentral source relative power, linear mixed models were computed with between-subject factor group (cTBS_SMA_, cTBS_SHAM_) and within-subject factor frequency band (delta, theta, alpha, beta, and low gamma), along with the interaction between group and frequency bands. Post hoc comparisons were performed using the Fisher LSD test with appropriate Bonferroni corrections. All the statistical analyses described thus far in this section were carried out using SPSS software (SPSS version 21, SPSS Inc.; Chicago, IL, USA).

## 3. Results

### 3.1. Across-Task Shared EEG Sources

**Figure 2:**
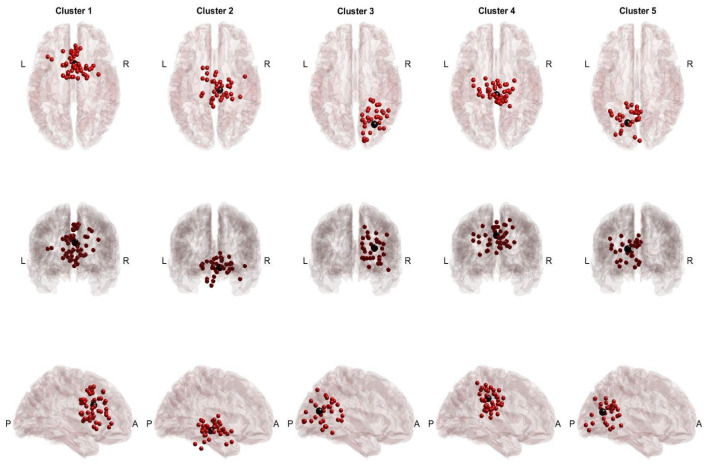
cTBS_SHAM_ cross-task dipoles and centroids. Red dots represent equivalent dipoles, and black dots represent the cluster centroid. Each cluster is in a column with superior, posterior, and lateral views.

**Figure 3:**
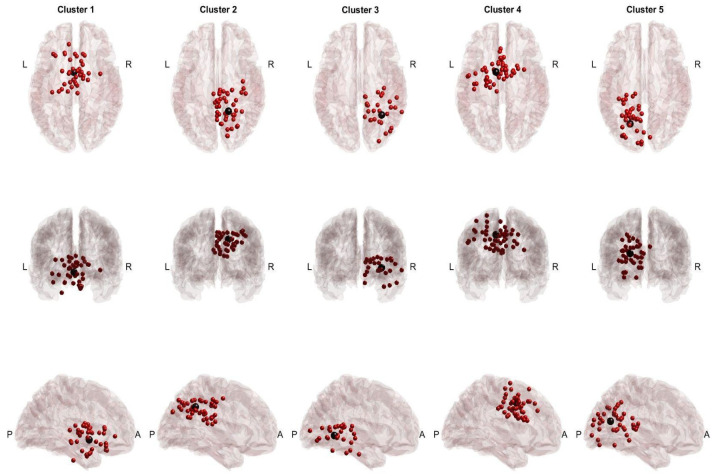
cTBS_SMA_ cross-task dipoles and centroids. Red dots represent equivalent dipoles, and black dots represent the cluster centroid. Each cluster is in a column with superior, posterior, and lateral views.

Twenty participants completed the protocol, ten participants received sham cTBS (cTBS_SHAM_), and another ten participants received cTBS (inhibitory) over left SMA (cTBS_SMA_) before the performance of SRT and PT. After preprocessing and artifactual source removal, the equivalent dipoles of the remaining sources were clustered using information from SRT and PT (cTBS_SHAM_, Figure 2; cTBS_SMA_, Figure 3), and the participant groups were computed separately. For the cTBS_SHAM_ group, common sources across tasks were found in the Cingulate Gyrus (BA 23 and 24) and the visual cortex (BA 19). In the cTBS_SMA_ group, common sources included the SMA (BA 6) and the visual cortex. Because the study region of interest was the frontocentral region, a cluster in the ventral anterior cingulate (Talairach [x,y,z]: [2,10,24]) for the cTBS_SHAM_ group and a cluster in the SMA region (Talairach [x,y,z]: [−9,1,41]) for the cTBS_SMA_ group was selected as clusters of interest for further analysis.

### 3.2. Relative Power Comparison within Cross-Task Clusters

**Figure 4:**
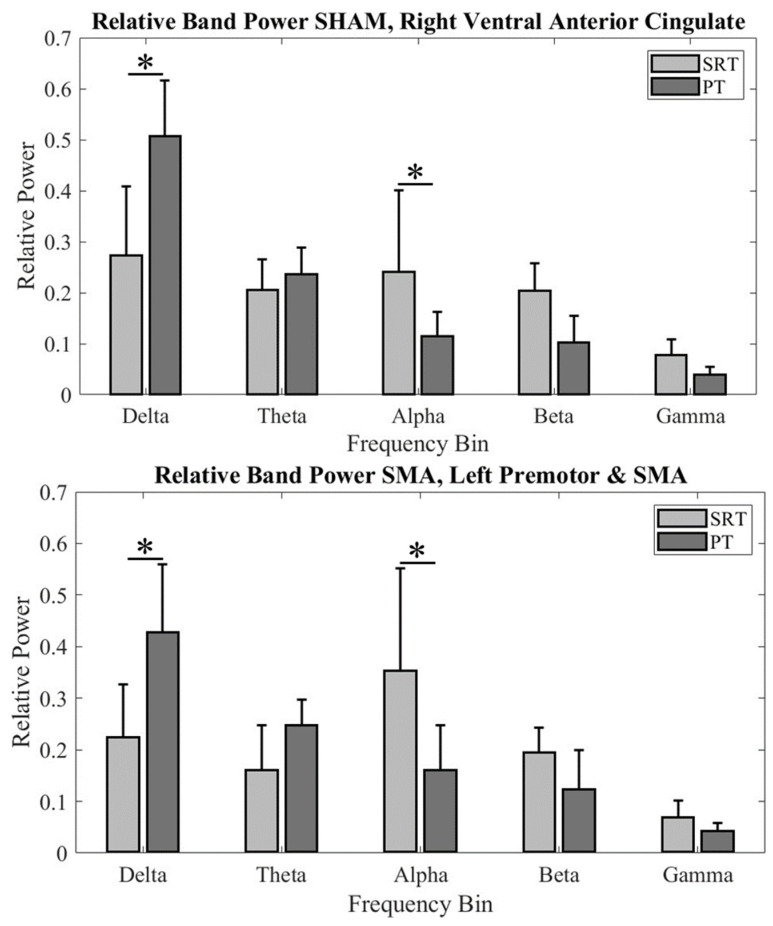
Band relative power across participants for (**Top**) cTBS_SHAM_ group Cluster 1 and (**Bottom**) cTBS_SMA_ group Cluster 4. An * denotes statistical significance of *p* < 0.05.

Relative EEG power was compared at different frequency bands between tasks within each group’s frontocentral cluster (Figure 4). For the ventral anterior cingulate (BA 24) in the cTBS_SHAM_ group, a significant difference was found in relative EEG power across two tasks (significant Task × Frequency bands interaction: F_4, 94.352_ = 21.352; *p* < 0.001; main effect of Frequency bands: F_4, 94.352_ = 60.958; *p* < 0.001). Post hoc comparisons found significantly higher delta-band power (*p* < 0.000001) and lower alpha-band power (*p* = 0.0098) during PT when compared to SRT. No other comparisons were found to be significant (all *p* values > 0.05).

Similarly, for the SMA (BA6) region in the cTBS_SMA_ group, a significant difference was found in relative EEG power across two tasks (significant Task × Frequency bands interaction: F_4, 94.352_ = 9.537; *p* < 0.001). Post hoc comparisons found significantly higher delta-band power (*p* < 0.00022) and lower alpha-band power (*p* = 0.00052) during PT when compared to SRT. No other comparisons were found to be significant (all *p* values > 0.05).

### 3.3. Frontocentral Activation during PT Across-Group Localization

**Figure 5:**
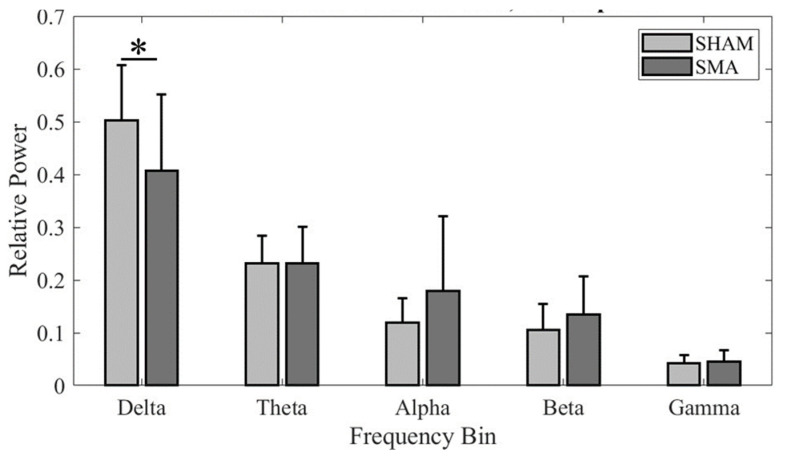
PT band relative power across groups for frontocentral cluster dipoles. An * denotes statistical significance of *p* < 0.05.

Since the secondary aim was to investigate the effects of cTBS over SMA on the EEG activity in the frontocentral region during PT, the analysis was focused on clusters formed using PT-related ICs in this region for each cTBS_SMA_ and cTBS_SHAM_ group. The Talairach coordinates of the cluster centroids in the frontocentral region were (0, 12, 42) for the cTBS_SHAM_ group, which suggests activation within BA 6 (SMA), and (2, −2, 48) for the cTBS_SMA_ group, which suggests activation within BA 6 (SMA). Band relative powers were computed for each IC from a participant contributing to these clusters and compared across groups (Figure 5). A significant difference was found in frequency band power across the two groups (significant Group × Frequency bands interaction: F_4, 195.187_ = 3.932; *p* = 0.004; main effect of Frequency bands: F_4, 195.187_ = 114.41; *p* < 0.001). Post hoc comparison found a significantly lower delta-frequency band power following cTBS over SMA (cTBS_SMA_) when compared with the cTBS_SHAM_ group (*p* = 0.038). No other comparisons were found to be significant (all *p* values > 0.05).

## 4. Discussion

This study compared EEG relative band power in the frontocentral region between two commonly studied, challenging balance tasks, SRT and PT, in neurotypical adults. The study also investigated whether cTBS over SMA can alter the EEG activations during PT in neurotypical adults. The novel findings from this study are (1) higher delta-band relative power and lower alpha-band relative power in the frontocentral region during PT when compared to SRT in both groups and (2) lower delta-band relative EEG power over the frontocentral region following cTBS over SMA when compared with sham stimulation.

In the study, two groups of individuals performed both the perturbation task and the sway reference task. Identified EEG sources of activations had commonalities and differences in the location of EEG clusters between the two groups. Mainly, EEG clusters localized within the frontocentral regions (cingulate gyrus, SMA), posterior cingulate cortex (PCC; BA 23 and 31), and visual cortex (VC; BA 17, 18 and 19), a finding consistent with previous studies [16,17,18,21,60]. However, the locations of these clusters identified in the two groups were distinct. For example, in the cTBS_SHAM_ and cTBS_SMA_ groups, the analysis found a cluster in the frontocentral regions, but these clusters were localized within the ventral anterior cingulate and supplementary motor area, respectively. As the purpose of this study was to compare EEG activations between two tasks, within-group analysis was performed for each cluster.

### 4.1. Lower Alpha-Band Relative Power during PT When Compared to SRT

A significant decrease in the alpha frequency band was found in the frontocentral region for PT as compared to SRT in both groups. Lower alpha frequency spectral power during a standing balance task has been argued to reflect higher task difficulty, i.e., more challenging balance task [10,14,18,61,62]. For example, Kahya et al. [62] increased difficulty and diverted attention through a standing balance task with mental arithmetic and found a decrease in alpha-band power as compared to quiet standing [62]. Lower alpha power during the performance of PT than SRT might be related to increased information processing necessary to process and/or respond to the platform translation resulting from reduced inhibition by higher centers [10,61,63,64].

### 4.2. Higher Delta-Band Relative Power during PT When Compared to SRT

A significant increase in the delta frequency band relative power was found during PT as compared to SRT in the frontocentral region for both groups. Delta oscillations are involved in cognitive processes such as decision-making and attentional processes [65] and are known to impact behavioral outcomes [66], where the difficulty of a postural task has been associated with increased delta band activity [67]. Slower brain oscillations due to their longer temporal window of processing information suggest the involvement of neurons in widespread brain areas [68,69]. Primate work has shown interactions between parietal sensory areas and motor areas in the frontal region in the delta frequency range during a somatosensory discrimination task [68]. The presence of delta band power in the frontocentral brain region during both balance tasks might represent long-range integrative processes with greater engagement of cognitive resources [70] during PT (i.e., greater delta band power) than SRT. Therefore, it is conceivable that greater task demand and challenge to the standing balance led to significantly higher delta-band power for PT than SRT.

### 4.3. Relation between Brain Oscillations and the Cortical Potentials following Balance Perturbations

The superimposition of oscillations in lower-frequency bands such as delta, theta, and alpha frequency bands may partly underlie the generation of cortical event-related potentials following balance perturbations [71,72,73,74,75]. Imposed mechanical perturbations such as the one delivered in PT are associated with characteristic event-related potentials (ERPs) in the EEG activity. Mainly, a positive potential (P1) is generated over the parietal-central region within the first 50–70 ms after the onset of perturbation. This response is followed by a large negative potential (N1) generated over the frontocentral region with a peak latency of 100–200 ms after the onset of perturbation [21,31,76,77]. This N1 response has been suggested to indicate the involvement of higher-order processing in the form of error detection (i.e., the difference between the actual balance state due to balance perturbation and the anticipated balance state) and signaling postural responses to the destabilizing effects of balance perturbations [12,31,77]. The size of N1 potential is small during SRT where sensory feedback regarding motor outputs during ongoing balance control is altered such that the effects are a function of the action of the participant [33]. Although the presence of a P1 response during SRT is debatable, our earlier work has shown the involvement of bilateral posterior parietal cortices during SRT [18]. The N1 response is followed by late evoked responses, the P2, and the N2 responses. There is a debate on the role of these late responses, with some studies suggesting their involvement in cortical sensorimotor processing while others linking them with a shift in attention to novel perturbation events [30,78,79]. In addition, the delta band power increases while the alpha band power decreases in response to stimulus and task demands [80,81]. Therefore, larger relative delta band power (and smaller relative alpha power) for PT, when compared with SRT, might explain larger and patterned ERPs observed following platform translation perturbations [80,81].

### 4.4. cTBS over SMA Altered Delta-Band Power over the Frontocentral Region during the PT Task

A cortical response in the fronto-parietal region is a well-documented phenomenon following mechanical perturbations of the standing platform (viz. translation). The clustering of frontocentral independent components for each participant showed activations of the SMA region (BA 6) for both groups. The study was designed to consider all trials together for clustering to provide a better understanding of the changes in cortical activity measured as EEG power in five frequency bands following inhibitory cTBS protocol. A significant decrease in the delta-frequency relative band power was found within the SMA region following cTBS over SMA when compared to sham stimulation. Previously, EEG frequency analysis of participants at rest reported significantly decreased power in the delta band following cTBS [82,83]. The results support this and report its occurrence during a standing balance task. cTBS might have increased neural noise in the stimulated area, thus disrupting the synchronicity of neural activities [84,85,86]. The reduction in delta-frequency-band relative power within the SMA following cTBS might thus suggest a disruption in long-range integrative processes between frontal and parietal regions necessary for the performance of PT [16,17,18,19,20,21,22,23,24]. The spread of TMS stimuli to distant regions through cortico–cortical connections is well known [87,88]. The fronto-parietal regions are known to have long-range cortico–cortical connections with Broadman area 4, premotor areas, cingulate areas, and parietal cortices [89,90]. Also, the smaller amplitude of delta oscillations following cTBS might have influenced the generation of ERPs in the frontocentral region, affecting the functional postural response following platform translation. However, this remains to be known. The study included only three trials per perturbation condition, thus limiting the statistical power to investigate the effects of cTBS over SMA on the amplitude and latency of ERPs and behavioral implications [91]. The study raises the possibility of using cTBS to alter ERP components by interfering with the underlying oscillations.

Our previous work using the sway reference task [18] found changes in EEG band power within bilateral parietal cortices and cingulate gyrus following cTBS over SMA, a frontocentral region, when compared with sham stimulation. Although this earlier study did not examine the effects of cTBS on delta-band power, it found changes in theta- and alpha-frequency band power following cTBS than sham stimulation. The changes in theta-band power over the frontocentral region following cTBS in our previous study were primarily observed for tasks with a lower level of difficulty but not for a high level of difficulty [18]. The theta-band power is known to not modulate consistently with changes in the balance task difficulty [61]. If there was any effect in the theta-frequency band in our study, it might have been masked due to data averaging across perturbation conditions that entailed varying levels of difficulty. The lack of an alpha effect in this study might be due to already lower alpha band power during PT (see Figure 4) and the inability of cTBS to lower it further, i.e., a floor effect.

## 5. Conclusions

Our findings suggest that PT is a more challenging balance task when compared with SRT, requiring greater cognitive function and attentional control, as indicated by higher delta and lower alpha relative frequency power in the frontocentral region. These findings provide a basic understanding of differences in the frontocentral activation between the two studied tasks in able-bodied individuals. This knowledge will lay the groundwork to understand the cross-task cortical changes due to aging and neurological conditions. Because task difficulty influences motor learning [92,93,94], our finding of differences in task difficulty between PT and SRT will have implications for how older adults and patients with neurological conditions relearn balance during rehabilitation. cTBS modulated delta power in the frontocentral cortical regions during PT. Additional research is needed to determine whether interference with delta oscillations affects the ERPs following platform perturbations. By isolating circuits that exhibit a favorable response to cTBS intervention, we will advance our understanding of precise brain circuits associated with balance control. Mechanistic findings may lead to cortical targets for effective neuromodulation strategies for rehabilitation of balance control in clinical populations.

## Data Availability

The processed data presented in the study are openly available in Mendeley Data at https://doi.org/10.17632/vdk5yr2m85.1.
